# Factors Associated With Highest Symptoms of Anxiety During COVID-19: Cross-Cultural Study of 23 Countries

**DOI:** 10.3389/fpsyg.2022.805586

**Published:** 2022-05-19

**Authors:** Valentina N. Burkova, Marina L. Butovskaya, Ashley K. Randall, Julija N. Fedenok, Khodabakhsh Ahmadi, Ahmad M. Alghraibeh, Fathil Bakir Mutsher Allami, Fadime Suata Alpaslan, Mohammad Ahmad Abdelaziz Al-Zu’bi, Kholoud Imhammad Meqbel Al-Mseidin, Derya Fatma Biçer, Hakan Cetinkaya, Oana Alexandra David, Silvia Donato, Seda Dural, Paige Erickson, Alexey M. Ermakov, Berna Ertuğrul, Emmanuel Abiodun Fayankinnu, Maryanne L. Fisher, Fakir Al Gharaibeh, Lauren Hocker, Ivana Hromatko, Elena Kasparova, Alexander Kavina, Yahya M. Khatatbeh, Hareesol Khun-Inkeeree, Kai M. Kline, Fırat Koç, Vladimir Kolodkin, Melanie MacEacheron, Irma Rachmawati Maruf, Norbert Meskó, Ruzan Mkrtchyan, Poppy Setiawati Nurisnaeny, Oluyinka Ojedokun, Damilola Adebayo, Mohd S. B. Omar-Fauzee, Barıs Özener, Edna Lúcia Tinoco Ponciano, Muhammad Rizwan, Agnieszka Sabiniewicz, Victoriya I. Spodina, Stanislava Stoyanova, Nachiketa Tripathi, Satwik Upadhyay, Carol Weisfeld, Mohd Faiz Mohd Yaakob, Mat Rahimi Yusof, Raushaniia I. Zinurova

**Affiliations:** ^1^Center of Cross-Cultural Psychology and Human Ethology, Institute of Ethnology and Anthropology, Russian Academy of Sciences, Moscow, Russia; ^2^International Centre of Anthropology, National Research University Higher School of Economics, Moscow, Russia; ^3^Counseling and Counseling Psychology, Arizona State University, Tempe, AZ, United States; ^4^Behavioral Sciences Research Center, Baqiyatallah University of Medical Sciences, Tehran, Iran; ^5^Department of Psychology, King Saud University, Riyadh, Saudi Arabia; ^6^Physical Education and Sport Sciences, University of Misan, Amarah, Iraq; ^7^Department of Anthropology, Istanbul University, Istanbul, Turkey; ^8^Department of Early Childhood, Faculty of Educational Sciences, Zarqa University, Zarqa, Jordan; ^9^Department of Educational and Psychological Counselling, Irbid National University, Irbid, Jordan; ^10^Department of Business Administration, Sivas Cumhuriyet University, Sivas, Turkey; ^11^Department of Psychology, Faculty of Human and Social Sciences, Yaşar University, İzmir, Turkey; ^12^Department of Clinical Psychology and Psychotherapy, Babes-Bolyai University, Cluj-Napoca, Romania; ^13^Department of Psychology, Università Cattolica del Sacro Cuore, Milan, Italy; ^14^Department of Psychology, İzmir University of Economics, İzmir, Turkey; ^15^Department of Psychology, University of Detroit Mercy, Detroit, MI, United States; ^16^Faculty of Bioengineering and Veterinary Medicine, Don State Technical University, Rostov-on-Don, Russia; ^17^Department of Sociology, Adekunle Ajasin University, Akungba, Nigeria; ^18^Department of Psychology, Saint Mary’s University, Halifax Regional Municipality, NS, Canada; ^19^Research Institute of Humanities and Social Sciences and Department of Sociology, University of Sharjah, Sharjah, United Arab Emirates; ^20^Department of Psychology, University of Zagreb, Zagreb, Croatia; ^21^Department of Pedagogy and Problems of Education Development, Belarusian State University, Minsk, Belarus; ^22^Department of History, St John’s University of Tanzania, Dodoma, Tanzania; ^23^Department of Psychology, Imam Mohammad Ibn Saud Islamic University, Riyadh, Saudi Arabia; ^24^Faculty of Education, Prince of Songkla University, Hat Yai, Thailand; ^25^Department of Anthropology, Hitit University, Çorum, Turkey; ^26^Faculty of Media Communications and Multimedia Technologies, Don State Technical University, Rostov-on-Don, Russia; ^27^School of Social & Behavioral Sciences, Arizona State University, Tempe, AZ, United States; ^28^Pasundan University, Bandung, Indonesia; ^29^Department for General and Evolutionary Psychology, Institute of Psychology, University of Pécs, Pécs, Hungary; ^30^Department of Cultural Studies, Faculty of History, Yerevan State University, Yerevan, Armenia; ^31^State Intelligence College, Bogor, Indonesia; ^32^Department of Pure & Applied Psychology, Adekunle Ajasin University, Akungba, Nigeria; ^33^School of Education, Universiti Utara Malaysia, Sintok, Malaysia; ^34^Institute of Psychology, University of the State of Rio de Janeiro, Rio de Janeiro, Brazil; ^35^Centre for Social Studies, University of Coimbra, Coimbra, Portugal; ^36^Department of Psychology, The University of Haripur, Haripur, Pakistan; ^37^Department of Otorhinolaryngology, Smell and Taste Clinic, TU Dresden, Dresden, Germany; ^38^Department of History and Ethnology, Ob-Ugric Institute of Applied Researches and Development, Pasundan University, Bandung, Indonesia; ^39^Department of Psychology, South-West University “Neofit Rilski”, Blagoevgrad, Bulgaria; ^40^Department of Humanities and Social Sciences, Indian Institute of Technology Guwahati, Guwahati, India; ^41^Institute of Innovation Management, Kazan National Research Technological University, Kazan, Russia

**Keywords:** anxiety, COVID-19, cross-cultural, personal experience, personal awareness, personal trust in official sources

## Abstract

The COVID-19 restrictions have impacted people’s lifestyles in all spheres (social, psychological, political, economic, and others). This study explored which factors affected the level of anxiety during the time of the first wave of COVID-19 and subsequent quarantine in a substantial proportion of 23 countries, included in this study. The data was collected from May to August 2020 (5 June 2020). The sample included 15,375 participants from 23 countries: (seven from Europe: Belarus, Bulgaria, Croatia, Hungary, Italy, Romania, Russia; 11 from West, South and Southeast Asia: Armenia, India, Indonesia, Iran, Iraq, Jordan, Malaysia, Pakistan, Saudi Arabia, Thailand, Turkey; two African: Nigeria and Tanzania; and three from North, South, and Central America: Brazil, Canada, United States). Level of anxiety was measured by means of the 7-item Generalized Anxiety Disorder Scale (GAD-7) and the 20-item first part of The State-Trait Anxiety Inventory (STAI)—State Anxiety Inventory (SAI). Respondents were also asked about their personal experiences with COVID-19, attitudes toward measures introduced by governments, changes in attitudes toward migrants during a pandemic, family income, isolation conditions, etc. The factor analysis revealed that four factors explained 45.08% of variance in increase of anxiety, and these components were interpreted as follows: (1) personal awareness of the threat of COVID-19, (2) personal reaction toward officially undertaken measures and attitudes to foreigners, (3) personal trust in official sources, (4) personal experience with COVID-19. Three out of four factors demonstrated strong associations with both scales of anxiety: high level of anxiety was significantly correlated with high level of personal awareness of the threat of COVID-19, low level of personal reaction toward officially undertaken measures and attitudes to foreigners, and high level of presence of personal experience with COVID-19. Our study revealed significant main effects of sex, country, and all four factors on the level of anxiety. It was demonstrated that countries with higher levels of anxiety assessed the real danger of a pandemic as higher, and had more personal experience with COVID-19. Respondents who trusted the government demonstrated lower levels of anxiety. Finally, foreigners were perceived as the cause of epidemic spread.

*“We have realized that we are on the same boat, all of us fragile and disoriented, but at the same time important and needed, all of us called to row together, each of us in need of comforting the other. On this boat. are all of us.*”

       Pope Francis, 2020

## Introduction

The COVID-19 pandemic has been a global challenge and has come to change the population’s daily life. Data using a sample of adults from different countries from 2020 to 2021 showed that social isolation, loneliness, and limitations are associated with worse mental and physical health (Berta et al., 2020; Brooks et al., 2020; Cao C. et al., 2020; Chen et al., 2020; Kowal et al., 2020; Mækelæ et al., 2020; van Bavel et al., 2020; Burkova et al., 2021; Butovskaya et al., 2021; Rodríguez et al., 2021; etc.). The negative psychological impact of the epidemic was demonstrated on the general population, as well as on children and the elderly (Cao C. et al., 2020; Chen et al., 2020; Fedenok and Burkova, 2020; Li et al., 2020; Yang et al., 2020). The negative psychological impact of the epidemic was demonstrated also in specific populations, i.e., health care workers (Zhang et al., 2020; Brailovskaia et al., 2021; Mansueto et al., 2021).

Studies observing the impact of epidemics have shown that a significant part of the population is subject to anxiety due to health threats and people’s desire to protect themselves and their loved ones (Jones and Salathe, 2009; Main et al., 2011; Jalloh et al., 2018; Bults et al., 2020; Burkova et al., 2021; Butovskaya et al., 2021; Semenova et al., 2021; Uehara et al., 2021). Past epidemics have shown that during their long pandemic (including quarantine) we are dealing with prolonged stress that can lead to immune system dysregulation and increased susceptibility to viral infections (Cohen et al., 2012), psychological distress and diagnostic symptoms of post-traumatic stress disorder (Reynolds et al., 2008; Taylor et al., 2008; Berta et al., 2020), depression and greater levels of stress (DiGiovanni et al., 2004; Hawryluck et al., 2004; Mak et al., 2009; Burkova et al., 2021; Rodríguez et al., 2021), insomnia, irritability, and low mood (Lee et al., 2005), and emotions of nervousness, fear, sadness, and guilt (Reynolds et al., 2008). Data from China confirm the high prevalence of post-traumatic stress disorder among the survivors of COVID-19 (Bo et al., 2020) and mental illness among the general population (Gao et al., 2020).

Scientists from different countries want to understand how the population responds to the social conditions imposed by the new coronavirus pandemic. A significant pool of studies from different countries showed the impact of the pandemic on increased anxiety, depression, post-traumatic stress, and even suicides (for example, Canada – Nwachukwu et al., 2020; Best et al., 2021; China – Bo et al., 2020; Cao C. et al., 2020; Gao et al., 2020; Huang and Zhao, 2020; etc.; France – Chaix et al., 2020; Husky et al., 2020; Greece – Voitsidis et al., 2020; Italy – Mazza et al., 2020; Japan – Tanoue et al., 2020; Malaysia – Kassim et al., 2021; Russia – Karpenko et al., 2020; Zinchenko et al., 2021; Spain – González-Sanguino et al., 2020; Rodríguez et al., 2021; United States – Czeisler et al., 2020; Khubchandani et al., 2021; etc.). The negative effects of COVID-19 on human psychological wellbeing and mental states worldwide have been demonstrated in more than 21,600 papers recently published according to the platform Scholar-google. This concerns both the stress associated with fear of illness (Abuhammad et al., 2021; Koçak et al., 2021; Luo et al., 2021), as well as governmental measures undertaken to stop the epidemic, such as lockdowns, social distancing, threat of or actual job loss and reduction of general internal and international mobility, etc. (Berta et al., 2020; Brooks et al., 2020; Fedenok and Burkova, 2020; Limcaoco et al., 2020; Mækelæ et al., 2020).

During 2020--2021, a number of cross-cultural studies were released that make a significant contribution to the understanding of major stress factors in different cultures^1^ (Berta et al., 2020; Kowal et al., 2020; Limcaoco et al., 2020; Mækelæ et al., 2020; Burkova et al., 2021; Butovskaya et al., 2021). The study of Limcaoco et al. (2020), which gathered data across 41 countries during the first wave of COVID-19 showed increasing levels of anxiety. Kowal et al. (2020) collected data from 26 countries and demonstrated associations of higher levels of stress from COVID-19 with younger age, being a single woman, lower level of education, staying with more children, and living in a country that has been severely affected by COVID-19. The same correlation of anxiety with younger age was found in our cross-cultural study conducted in 23 countries (Burkova et al., 2021). Mækelæ et al. (2020) assessed effectiveness of introduced restrictions, their impact on daily life, and general distress and paranoia during the first outbreak in five countries – Brazil, Colombia, Germany, Israel, Norway, and the United States. Participants from Brazil, Colombia, and the United States reported the highest level of distress, whereas people from Israel, Norway and Germany had comparatively lower levels of distress (Mækelæ et al., 2020). Data from Russia and Spain demonstrated that for the Russian sample’s perceived social support from the family was the only predictor for a reduced rate of anxiety, whereas for the Spanish sample it was social support from three sources: significant others, family, and friends (Berta et al., 2020). The same results were found among Chinese students – social support had a negative relationship with anxiety (Cao C. et al., 2020). Cross-cultural comparisons of psychosocial distress in the United States, South Korea, France, and Hong Kong during the initial phase of COVID-19 showed that younger age, greater concern for COVID-19, and more severe loneliness predicted worse psychological outcome; and the magnitudes of these effects varied across the four regions (Dean et al., 2021). The association between depression symptoms, psychological burden caused by COVID-19 and physical activity were found in Germany, Italy, Russia, and Spain – burden by COVID-19 was significantly positively associated with depression symptoms, while it was significantly negatively linked to physical activity, and physical activity buffered the association between depression symptoms and burden (Brailovskaia et al., 2021). Earlier it was demonstrated by our research team that cultural dimensions, such as individualism/collectivism, power distance and looseness/tightness may function as protective adaptive mechanisms against the development of anxiety disorders in a pandemic situation – participants from countries with the highest ratings of anxiety were also highest on individualism and looseness, and lowest ratings on power distance (Burkova et al., 2021). It was also revealed that factors of cohabitation/loneliness somehow produced different effects on anxiety in different countries. While in a majority of countries, people who lived with someone reported the highest level of aggression, in such countries as Belarus, Bulgaria, and Malaysia, whereas Pakistan showed the opposite effect (Burkova et al., 2021).

Despite a great number of studies, conducted on stress and distress, as well as coping strategies in the time of COVID-19, it remained far from being obvious, which cultural differences worsen the situation or on the contrary reduce the citizens’ anxiety. We have already demonstrated the gender differences in stress levels during the first wave of a pandemic in 23 countries, as well as the effects of age and living condition on decrease or increase of stress levels (Burkova et al., 2021). The goals of the present study are to examine possible factors that may be associated with self-reported levels of anxiety during the time of the first wave COVID-19 quarantine in a large sample from 23 countries. Also, we are planning to analyze the effects of personal awareness of the threat of COVID-19, personal reaction toward officially undertaken measures and attitudes to foreigners, personal trust toward official sources, and personal experience with COVID-19 on stress levels in a cultural-specific perspective.

## Materials and Methods

The survey was conducted during the first wave of the pandemic COVID-19 from May to August 2020 (Median 5 June 2020). According to the WHO, on this date worldwide there were registered 6,515,796 confirmed cases of COVID-19 and 387,298 confirmed deaths^2^ (see country details in Table 1). All coauthors collected data in their home countries for this study. The questionnaire was generated on the Google Forms service hosted by the principal investigator. The original questionnaire was developed in Russian and English. In all non-English speaking countries (except Russia), colleagues translated the measures into their native languages using a back-translation procedure (Sousa and Rojjanasrirat, 2011).

**TABLE 1:** Sample characteristics and distribution by country, sex, and age.

Country	Language	Total	Sex		Mean age	
	of survey		Male	Female	(±SD)	Total confirmed cases/death on 5 June 2020*
Armenia	Armenian	33	27	6	20.45 (±2.37)	11,817/183
Belarus	Russian	338	143	195	19.20 (±2.85)	45,981/253
Brazil	Portuguese	515	82	430	38.80 (±13.78)	584,016/32,548
Bulgaria	Bulgarian	322	129	193	28.34 (±8.75)	2,585/147
Canada	English	692	446	246	30.33 (±8.74)	93,441/7,543
Croatia	English	275	71	204	24.10 (±8.40)	2,247/103
Hungary	Hungarian	235	35	198	31.95 (±11.84)	3,954/539
India	English	383	213	170	29.95 (±9.85)	226,770/6,348
Indonesia	Indonesian	930	504	424	32.05 (±12.09)	28,818/1,721
Iran	Persian	306	88	217	33.68 (±7.34)	164,270/8,071
Iraq	Arabic	173	88	85	35.03 (±10.63)	8,840/271
Italy	Italian	253	44	208	23.50 (±4.15)	234,013/33,689
Jordan	Arabic	449	121	328	33.68 (±10.52)	765/9
Malaysia	Malay	1087	478	609	33.19 (±11.12)	8,247/115
Nigeria	English	316	214	102	34.09 (±11.24)	11 516/323
Pakistan	English	484	212	272	27.06 (±11.11)	89,249/1,838
Romania	Romanian	269	42	226	36.22 (±10.94)	19,907/1,299
Russia	Russian	1903	486	1417	20.99 (±4.72)	449,834/5,528
Saudi Arabia	Arabic	414	98	316	26.76 (±9.72)	93,157/611
Tanzania	English	341	185	156	23.95 (±4.25)	509/21
Turkey	Turkish	4717	1609	3093	27.57 (±10.84)	167,410/4,630
Thailand	Thai	300	49	250	32.82 (±13.00)	3,102/58
United States	English	666	189	477	45.16 (±17.15)	1,837 803/106,876
**Total**		**15375**	**5553**	**9822**	**29.15 (±11.80)**	

Participants in each country were recruited from various university listservs and social networking sites. Inclusion criteria were: (1) being more than 18 years of age; (2) responding no to having a chronic disease and/or predisposition for depression or having received treatment (based on self-assessments of participants). People with chronic diseases and a predisposition to/or depression/treatment were excluded from the sample, as such respondents already have an increased level of anxiety due to illness/depression, and it would be more difficult to isolate the influence of COVID factors. If eligible, participants were directed to complete the self-report survey on Google forms to provide informed consent, and were asked to take a survey, described below, which took approximately 20 min to complete. Participants were not compensated for their participation.

The study was conducted according to the principles expressed in the Declaration of Helsinki. The Scientific Council of the Institute of Ethnology and Anthropology of the Russian Academy of Sciences (protocol No01, dated April 9, 2020) approved the protocols used to recruit participants and to collect data before conducting this study. All participants provided written informed consent before completing the survey.

The sample is made up of 15,375 participants from 23 countries (7 European: Belarus, Bulgaria, Croatia, Hungary, Italy, Romania, Russia; 11 Asian: Armenia, India, Indonesia, Iran, Iraq, Jordan, Malaysia, Pakistan, Saudi Arabia, Thailand, Turkey; 2 African: Nigeria and Tanzania; and 3 from North, South, and Central America: Brazil, Canada, United States). The mean age of the total sample was 29 years old and mean scores of ages in each country are presented in Table 1.

The variables and instruments included in the assessment were the following:

*Sociodemographic information*: sex, region, marital status, number of children, religion, place of residence, age, origin, educational level, family income, and chronic diseases.

*Variables related to COVID-19:* personal experiences with COVID-19, reaction toward measures introduced by governments, changes in attitudes toward migrants during a pandemic, isolation conditions, etc. (see questions in Table 2).

**TABLE 2:** Factor loadings for the 12 questions about personal experiences with COVID-19 and conditions in total sample.

Questions	Factor loadings			
	*PC1:* personal awareness of the threat of COVID-19	PC2: personal reaction toward officially undertaken measures and attitudes to foreigners	PC3: personal trust to official sources	PC4: personal experience with COVID-19
Do you think the coronavirus pandemic poses a real threat for you personally? 0 = NO, 1 = YES	0.749			
Do you think the coronavirus pandemic poses a real threat for your relatives? 0 = NO, 1 = YES	0.692			
Do you have COVID-19 infected people in your close environment? 0 = NO, 1 = YES				0.633
Have you been diagnosed with COVID-19? 0 = NO, 1 = I have had symptoms, but have not been tested, 2 = YES				0.594
Do you include in risk group of COVID-19 (returned from countries unfavorable for epidemic situations, had close contact with patients?) 0 = NO, 1 = YES				0.725
Has your family income changed after restrictions during COVID-19? 1 = DECREASED, 2 = NOT CHANGE, 3 = INCREASED			0.329	
Have you become more hostile and suspicious toward foreigners (total)? 0 = NO, 1 = YES		0.549		
Are the actions of the authorities on the regime of self-isolation legitimate? 0 = NO, 1 = YES			0.736	
Are these measures, undertaken by authorities on the lock down, self-isolation sufficient? 0 = NO, 1 = YES		0.713		
Are these measures introduced: too early? in time? too late?		–0.673		
Do you trust information coming from official sources (i.e., the government)? 0 = NO, 1 = YES			0.719	

*Anxiety measurements*: two questionnaires for measurement of anxiety level were used in this study - Generalized Anxiety Disorder Scale (GAD-7) created by Spitzer et al. (2006) and State Anxiety Inventory (SAI) created by Spielberger (1983). We chose two scales of anxiety, because each of them targets different aspects of this phenomenon. GAD-7 screens for the presence of anxiety and related disorders (difficulties in controlling concerns, restlessness, mild fatigue, difficulty concentrating, irritability, muscle tension and sleep problems), while SAI evaluates anxiety as a reaction to stress (“in the moment” anxiety). Validated measures of the GAD-7 and SAI were used when available (Hanin and Spielberger, 1980; Sipos and Sipos, 1980; Spielberger, 1983; Spitzer et al., 2006; Sidik et al., 2012; Bozukluğu et al., 2013; Bahammam Maha, 2016; Esipenko et al., 2018; Musumari et al., 2018; Silva et al., 2018; Dzhambov et al., 2019; Al-Rabiaah et al., 2020).

The GAD-7 consists of seven items based on seven main symptoms and examines their frequency over the past 2 weeks (Toussaint et al., 2020). Respondents report their symptoms using a 4-point Likert rating scale ranging from 0 (not at all) to 3 (almost every day) with a total score ranging from 0 to 21. Total scores across the seven items were calculated, and anxiety symptoms were classified as norm (0–4), mild (5–9), moderate (10–14), and severe (15–21) (Toussaint et al., 2020). Alpha reliability coefficients in the present study for GAD-7 were 0.895.

Anxiety as an emotional state was measured with the first part of The State-Trait Anxiety Inventory (STAI) – State Anxiety Inventory (SAI). It consists of a 20-item scale for measuring the intensity of anxiety as an emotional state. People report the intensity of their feelings of anxiety right now, at this moment by rating themselves on the following 4-point Likert scale from 1 (not at all) to 4 (very much so). Total scores of anxiety symptoms were classified as norm/low (0–30), moderate (31–45), and high (46 and above) (Spielberger, 1983). Alpha reliability coefficients in the present study for SAI were 0.766.

### Data Analysis

SPSS (Version 27.0) was employed for data evaluation. Data was evaluated for missingness, and the final sample included those questionnaires in which sociodemographic information and anxiety scale responses were fully completed. The alpha reliability coefficient in the present study for GAD-7 was 0.90. The alpha reliability coefficient in the present study for SAI was 0.77.

An analysis of descriptive statistics was illustrating the country differences on anxiety scales. GLM ANOVA was used for analysis of the GAD-7 and SAI to estimate the association between sex and country on levels of anxiety. In order to explore the relationship between the questionnaires of this study and anxiety scales, factor analysis was used (factor analysis with Varimax rotation). The analysis included all questions for which loadings were higher than 0.30. We assessed statistically meaningful loadings by using the criteria of 0.32 (“poor”), 0.45 (“fair”), 0.55 (“good”), 0.63 (“very good”), and 0.71 (“excellent”) (Tabachnick et al., 2007). Linear regression was used to test the associations between the GAD-7, SAI scales and four factors.

## Results

### Country Differences on Anxiety Scales

**FIGURE 1:**
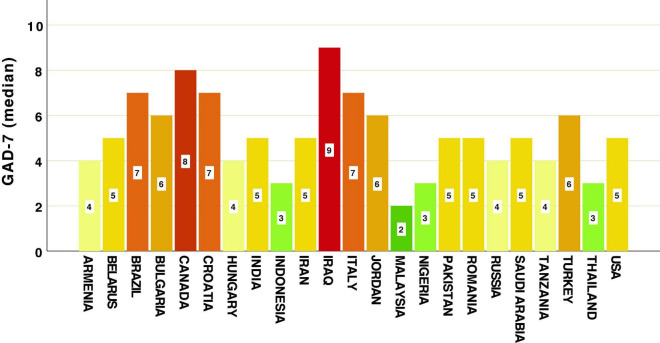
Country differences in levels of Generalized Anxiety Disorder Scale (GAD-7).

**FIGURE 2:**
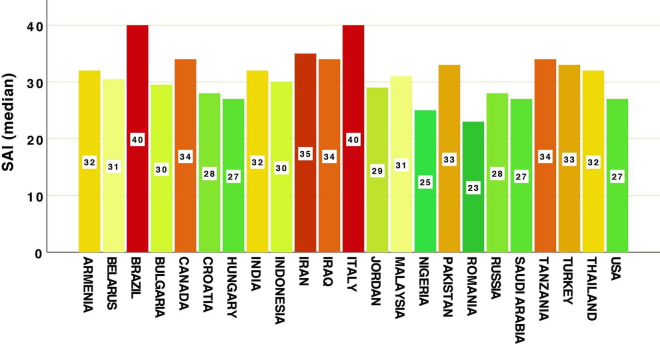
Country differences in levels of State Anxiety Inventory (SAI).

Means and medians of GAD-7 and SAI scores across countries are represented in Table 3 and Figures 1, 2. Our data revealed that the highest level of anxiety during restrictions and lockdown of the first wave of COVID-19 were in participants from Iraq, Canada, Brazil, Croatia and Italy when looking at the GAD-7 scale (Figure 1). Most of the highest levels of state anxiety (SAI) were in Brazil, Italy, and Iran (Figure 2). Lowest anxiety countries were Malaysia, Indonesia, Thailand (measured by GAD-7), Romania and Nigeria (measured by SAI) (Figures 1, 2 and Table 3).

**TABLE 3:** Descriptive statistics of GAD-7 and SAI scales by country.

Country		*N*	GAD-7 scale	SAI scale
			Mean (±SD)	Mean (±SD)
6.01	Armenia	33	5.48 (±4.95)	30.06 (±12.39)
	Belarus	338	5.89 (±4.60)	30.99 (±10.28)
	Brazil	515	8.43 (±5,73)	39.33 (±12.18)
	Bulgaria	322	6.74 (±4.76)	28.75 (±12.14)
	Canada	692	8.10 (±5.38)	31.83 (±10.70)
	Croatia	275	7.43 (±4.73)	28.32 (±12.10)
	Hungary	235	4.91 (±4.51)	28.19 (±12.18)
	India	383	6.00 (±4.94)	31.70 (±9.21)
	Indonesia	930	4.34 (±4.61)	28.33 (±10.95)
	Iran	306	5.71 (±4.36)	34.94 (±3.07)
	Iraq	173	9.16 (±4.95)	32.43 (±9.89)
	Italy	253	7.69 (±4.28)	38.44 (±10.88)
	Jordan	449	6.54 (±4.84)	28.35 (±10.78)
	Malaysia	1087	3.16 (±4.10)	28.19 (±10.31)
	Nigeria	316	4.40 (±4.85)	25.01 (±10.72)
	Pakistan	484	6.16 (±5.20)	30.73 (±11.72)
	Romania	269	5.52 (±4.67)	23.71 (±11.73)
	Russia	1903	5.22 (±4.91)	28.41 (±11.77)
	Saudi Arabia	414	5.52 (±4.64)	27.06 (±12.16)
	Tanzania	341	4.96 (±5.06)	32.80 (±6.01)
	Turkey	4717	6.86 (±4.90)	33.21 (±8.03)
	Thailand	300	4.09 (±4.12)	30.69 (±8.42)
	United States	666	6.33 (±5.42)	27.18 (±13.61)
	**Total**	**15375**	**6.04 (±5,039)**	**30.83 (±10.69)**

**FIGURE 3:**
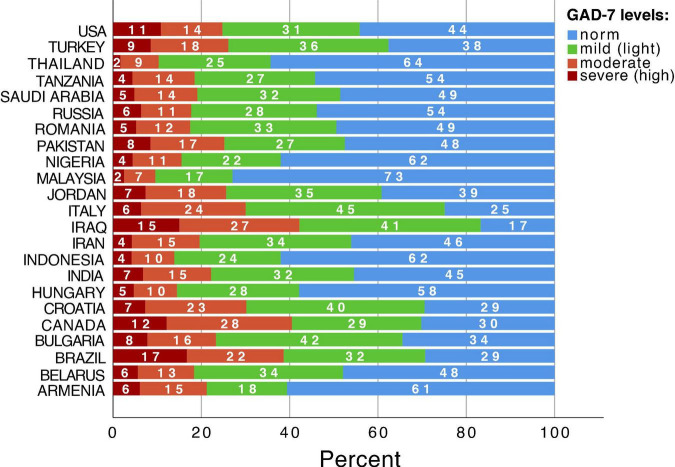
Country differences of levels of Generalized Anxiety Disorder Scale (GAD-7).

In the total sample 7045 participants (45.84%) had no symptoms of anxiety on GAD-7 (norm level), whereas people with mild anxiety were 31.43% (4830), moderate – 15.40% (2366), and severe – 7.33% (1127). Cross-cultural differences of levels of GAD-7 anxiety scales are demonstrated in Figure 3. The largest percentage of people with the highest levels of anxiety (red color) was in Brazil (17%), Iraq (15%), Canada (12%), and the United States (11%) (Figure 3). The lowest percentage of people with the highest levels of anxiety was in Malaysia (2%) and Thailand (2%) (Figure 3).

**FIGURE 4:**
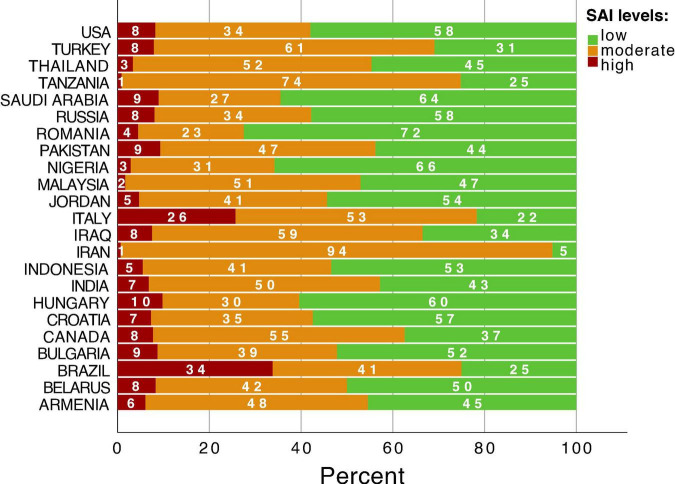
Country differences of levels of State Anxiety Inventory (SAI).

As for the level of state anxiety SAI, low values were observed in 43.08% (6589) of respondents, moderate – 49.42% (7560), and high – 7.50% (1147). Cross-cultural differences of levels of SAI anxiety scales are demonstrated in Figure 4. The largest percentage of people with the highest levels of anxiety (red color) were found in Brazil (34%) and Italy (26%) (Figure 4). The lowest percentage of people with the highest levels of anxiety were detected in Iran (1%) and Tanzania (1%) (Figure 4).

The results of GLM ANOVAs with GAD-7 as the dependent variable, sex and country as fixed factors and significant main effects of sex (*F*_1_,_15340_ = 298.885, *p* < 0.001, η^2^ = 0.019) and country (*F*_22_,_15345_ = 53.758, *p* < 0.001, η^2^ = 0.072), showed small and medium effect sizes accordingly. In the case of SAI as the dependent variable we found main effects of sex (*F*_1_,_15268_ = 157.504, *p* < 0.001, η^2^ = 0.010) and country (*F*_22_,_15273_ = 67.872, *p* < 0.001, η^2^ = 0.089), both with medium effect sizes. Sex differences across countries were already discussed in our early paper (see more details in Burkova et al., 2021).

### Anxiety Scales and Personal Experiences With COVID-19

In order to explore the relationship between the questionnaires of this study and anxiety scales, factor analysis was used (Varimax rotation). As seen in Table 2, the factor loadings of the ten items ranged between 0.55 and 0.75, suggesting that each item substantially contributes to the factor at good and excellent levels. The four factors explained 45.08% of total variance. The first factor (PC1), interpreted as personal awareness of the threat of COVID-19, accounted for 13.48% of variance. The second factor (PC2) explained 11.60% of variance and reflected personal reaction toward officially undertaken measures and attitudes to foreigners. The third factor (PC3) revealed that 10.47% of total variance was associated with personal trust in official sources. Finally, the fourth factor (PC4) explained 9.53% of variance and was interpreted as personal experience with COVID-19.

Three factors correlated significantly with both scales of anxiety; however, the correlations were not high, and this was especially evident for positive correlation between the level of anxiety and personal awareness of the threat of COVID-19 (PC1) (Table 4). High levels of anxiety were significantly correlated with low levels of personal trust in official sources (PC3) and with high levels of presence of personal experience with COVID-19 (PC4) (Table 4).

**TABLE 4:** Correlation analysis of the anxiety scales with control for country and sex and four factors in total sample.

Variables	PC1: personal awareness of the threat of COVID-19 r (p)	PC2: personal reaction towards officially undertaken measures and attitudes to foreigners r (p)	PC3: personal trust in official sources r (p)	PC4: personal experience with COVID-19 r (p)
GAD-7	0.054 (<0.001)	–0.004 (NS)	–0.119 (<0.001)	0.125 (<0.001)
SAI	0.168 (<0.001)	–0.039 (<0.001)	–0.118 (<0.001)	0.099 (<0.001)

*r, coefficient of correlation; p, significance; NS, not significant.*

The results of GLM ANOVA with GAD-7 as the dependent variable, sex and country as fixed factors, and four selected factors as covariates, revealed significant main effects of sex [*F*_(1)_ = 303.748, *p* = 2.3405E-67, $\eta_{p}^{2}$ = 0.020], country [*F*_(21)_ = 49.830, *p* = 8.0322E-201, $\eta_{p}^{2}$ = 0.066], and all factors – PC1 [personal awareness of the threat of COVID-19: *F*_(1)_ = 67.639, *p* = 2.1259E-16, $\eta_{p}^{2}$ = 0.005], PC2 [personal reaction toward officially undertaken measures and attitudes to foreigners: *F*_(1)_ = 16.289, *p* = 0.000055, $\eta_{p}^{2}$ = 0.001], PC3 [personal trust in official sources: *F*_(1)_ = 197.176, *p* = 1.6598E-44, $\eta_{p}^{2}$ = 0.013], and PC4 [personal experience with COVID-19: *F*_(1)_ = 113.777, *p* = 1.8172E-26, $\eta_{p}^{2}$ = 0.008], with small effect sizes.

The results of GLM ANOVA with SAI as the dependent variable, sex and country as fixed factors, and four selected factors as covariates, revealed significant main effects of sex [*F*_(1)_ = 154.202, *p* = 3.1302E-35, $\eta_{p}^{2}$ = 0.010], country [*F*_(21)_ = 58.630, *p* = 1.8862E-237, $\eta_{p}^{2}$ = 0.077], and all factors – PC1 [personal awareness of the threat of COVID-19: *F*_(1)_ = 234.853, *p* = 1,3168E-52, $\eta_{p}^{2}$ = 0.016], PC2 [personal reaction toward officially undertaken measures and attitudes to foreigners: *F*_(1)_ = 106.979, *p* = 5.4706E-25, $\eta_{p}^{2}$ = 0.007], PC3 [personal trust in official sources: *F*_(1)_ = 193.724, *p* = 9.211E-44, $\eta_{p}^{2}$ = 0.013], and PC4 [personal experience with COVID-19: *F*_(1)_ = 154.202, *p* = 3.1302E-35, $\eta_{p}^{2}$ = 0.010], with small effect sizes.

In the next step we estimated the relationship between anxiety scales and four factors using regression analysis. Significant linear effects on GAD-7 were demonstrated with PC1, PC3, and PC4 in the total sample (Table 5). Countries with high levels of anxiety assessed the more real personal awareness of the threat of COVID-19 (PC1) and had more personal experience with COVID-19 (PC4). Low levels of anxiety were observed in those people who personally trusted official sources (PC3).

**TABLE 5:** Regression analysis for the factors predicting anxiety (GAD-7 as dependent variable, *R*^2^ = 0.032) in total sample.

Predictor	*B*	*SE*	Beta	*T*	*p*
*PC1: personal awareness of the threat of COVID-19*	0.275	0.041	0.055	6.795	<0.001
*PC2: personal reaction toward officially undertaken measures and attitudes to foreigners*	–0.058	0.041	–0.012	–1.435	NS
*PC3: personal trust in official sources*	–0.586	0.041	–0.116	–14.460	<0.001
*PC4: personal experience with COVID-19*	0.614	0.041	0.112	15.146	<0.001

*NS, not significant.*

Strong significant linear effects on SAI have been demonstrated for all four factors (Table 6). Personal trust in official sources (public trust that the measures introduced by government are sufficient and introduced in a timely manner) correlated significantly with lower self-reported anxiety. Also, personal reaction toward officially undertaken measures and attitudes to foreigners correlated significantly with lower self-reported anxiety.

**TABLE 6:** Regression analysis for the factors predicting anxiety (SAI as dependent variable, *R*^2^ = 0.053) in total sample.

Predictor	*B*	*SE*	Beta	*t*	*P*
*PC1: personal awareness of the threat of COVID-19*	1.800	0.086	0.167	20.960	<0.001
*PC2: personal reaction toward officially undertaken measures and attitudes to foreigners*	–0.464	0.086	–0.043	–5.410	<0.001
*PC3: personal trust in official sources*	–1.236	0.086	–0.115	–14.406	<0.001
*PC4: personal experience with COVID-19*	1.058	0.086	0.098	12.306	<0.001

The results of a regression analysis with GAD-7 as tested variable and the four factors as independent variables per each country are presented in Table 7. We excluded Tanzania from analysis, as some questions were not completed by respondents from this country.

**TABLE 7:** Regression analysis for the factors predicting anxiety (GAD-7) in each country.

Country	*R* ^2^	Predictor	*B*	*SE*	Beta	*t*	*p*
Armenia	0.329	PC1	1.454	0.809	0.283	1.798	0.083
		PC2	–0.532	0.897	–0.096	–0.593	0.558
		PC3	0.561	0.831	0.110	0.674	0.506
		PC4	3.414	1.060	0.511	3.221	**0.003**
Belarus	0.046	PC1	0.223	0.328	0.038	0.682	0.496
		PC2	0.227	0.387	0.032	0.588	0.557
		PC3	–0.994	0.297	–0.185	–3.353	**0.001**
		PC4	0.356	0.246	0.078	1.448	0.149
Brazil	0.058	PC1	0.540	0.394	0.062	1.372	0.171
		PC2	–0.312	0.363	–0.038	–0.862	0.389
		PC3	–1.027	0.249	–0.186	–4.119	**<0.001**
		PC4	0.290	0.156	0.080	1.854	0.064
Bulgaria	0.018	PC1	0.237	0.242	0.056	0.981	0.327
		PC2	–0.139	0.335	–0.024	–0.416	0.678
		PC3	0.050	0.261	0.011	0.190	0.849
		PC4	0.608	0.314	0.110	1.939	0.053
Canada	0.050	PC1	–0.138	0.232	–0.027	–0.593	0.553
		PC2	0.387	0.247	0.065	1.568	0.117
		PC3	–0.300	0.284	–0.050	–1.057	0.291
		PC4	0.711	0.151	0.195	4.694	**<0.001**
Croatia	0.072	PC1	1.158	0.316	0.216	3.669	**<0.001**
		PC2	–0.237	0.406	–0.035	–0.582	0.561
		PC3	–0.608	0.299	–0.121	–2.030	**0.043**
		PC4	–0.391	0.376	–0.062	–1.038	0.300
Hungary	0.080	PC1	0.875	0.299	0.188	2.928	**0.004**
		PC2	–1.181	0.377	–0.199	–3.135	**0.002**
		PC3	–0.268	0.320	–0.054	–0.838	0.403
		PC4	–0.185	0.298	–0.039	–0.620	0.536
India	0.056	PC1	0.539	0.262	0.105	2.061	**0.040**
		PC2	0.052	0.294	0.009	0.176	0.860
		PC3	–0.683	0.305	–0.120	–2.236	**0.026**
		PC4	0.819	0.270	0.152	3.039	**0.003**
Indonesia	0.060	PC1	0.544	0.168	0.104	3.235	**0.001**
		PC2	–0.238	0.187	–0.042	–1.269	0.205
		PC3	–1.123	0.200	–0.185	–5.622	**<0.001**
		PC4	0.345	0.164	0.067	2.098	**0.036**
Iran	0.069	PC1	0.376	0.262	0.082	1.432	0.153
		PC2	0.641	0.335	0.109	1.911	0.057
		PC3	–1.186	0.306	–0.220	–3.874	**<0.001**
		PC4	0.192	0.154	0.070	1.245	0.214
Iraq	0.033	PC1	–0.034	0.485	–0.006	–0.070	0.944
		PC2	–0.498	0.573	–0.074	–0.870	0.386
		PC3	–0.716	0.416	–0.148	–1.723	0.087
		PC4	0.020	0.384	0.004	0.052	0.958
Italy	0.026	PC1	0.460	0.297	0.099	1.548	0.123
		PC2	–0.514	0.357	–0.092	–1.438	0.152
		PC3	–0.132	0.357	–0.024	–0.371	0.711
		PC4	0.213	0.230	0.059	0.927	0.355
Jordan	0.036	PC1	0.689	0.188	0.176	3.668	**<0.001**
		PC2	0.244	0.386	0.031	0.631	0.528
		PC3	–0.574	0.286	–0.100	–2.011	**0.045**
		PC4	0.253	0.431	0.028	0.586	0.558
Malaysia	0.053	PC1	–0.516	0.179	–0.092	–2.880	**0.004**
		PC2	–0.325	0.231	–0.043	–1.410	0.159
		PC3	0.019	0.340	0.002	0.056	0.955
		PC4	1.678	0.257	0.196	6.538	**<0.001**
Nigeria	0.028	PC1	0.296	0.230	0.072	1.289	0.198
		PC2	0.192	0.383	0.028	0.502	0.616
		PC3	–0.610	0.270	–0.128	–2.264	**0.024**
		PC4	0.421	0.325	0.073	1.295	0.196
Pakistan	0.063	PC1	0.892	0.229	0.174	3.905	**<0.001**
		PC2	–0.238	0.302	–0.036	–0.788	0.431
		PC3	–0.787	0.246	–0.145	–3.202	**0.001**
		PC4	0.182	0.196	0.041	0.931	0.353
Romania	0.019	PC1	0.604	0.289	0.128	2.094	**0.037**
		PC2	–0.060	0.383	–0.010	–0.156	0.876
		PC3	–0.268	0.387	–0.043	–0.692	0.490
		PC4	0.156	0.264	0.036	0.590	0.556
Russia	0.036	PC1	0.256	0.109	0.056	2.363	**0.018**
		PC2	–0.414	0.143	–0.069	–2.895	**0.004**
		PC3	–0.502	0.105	–0.111	–4.787	**<0.001**
		PC4	0.646	0.141	0.104	4.570	**<0.001**
Saudi Arabia	0.122	PC1	1.156	0.204	0.268	5.670	**<0.001**
		PC2	–0.477	0.375	–0.059	–1.274	0.203
		PC3	–0.917	0.342	–0.127	–2.682	**0.008**
		PC4	0.730	0.304	0.112	2.399	**0.017**
Turkey	0.016	PC1	0.291	0.087	0.048	3.343	**0.001**
		PC2	–0.115	0.074	–0.024	–1.554	0.120
		PC3	–0.507	0.070	–0.113	–7.283	**<0.001**
		PC4	0.205	0.083	0.036	2.468	**0.014**
Thailand	0.016	PC1	–0.035	0.249	–0.008	–0.142	0.887
		PC2	–0.238	0.332	–0.044	–0.718	0.473
		PC3	–0.740	0.466	–0.099	–1.588	0.113
		PC4	0.273	0.302	0.053	0.905	0.366
United States	0.068	PC1	0.687	0.239	0.118	2.880	**0.004**
		PC2	–0.312	0.282	–0.045	–1.107	0.269
		PC3	–1.200	0.247	–0.186	–4.863	**<0.001**
		PC4	0.380	0.154	0.095	2.471	**0.014**

*The bold values show significant association.*

In the case of the first factor (PC1), 11 countries demonstrated a positive association between anxiety and personal awareness of the threat of COVID-19 (Croatia, Hungary, India, Jordan, Malaysia, Pakistan, Romania, Russia, Saudi Arabia, United States), meaning that a high levels of anxiety were registered for people with high levels of personal awareness of the threat of COVID-19. The exception was Malaysia. The Malayan respondents with significantly higher levels of GAD-7 were those who reported a low level of personal awareness of the threat of COVID-19 (Table 7).

The second factor (PC2) significantly predicted of anxiety only in two countries – Russia and Hungary. More anxious people in these countries were those who did not believe in officially undertaken measures and think that measures were introduced too late, as well as those who felt more hostile and suspicious reaction to foreigners (Table 7).

Personal trust in official sources (PC3) was the significant predictor of GAD-7 in 13 countries – Belarus, Brazil, Croatia, India, Indonesia, Iran, Jordan, Nigeria, Pakistan, Russia, Saudi Arabia, Turkey, and the United States. High levels of anxiety in these countries were associated with low personal trust in government and official sources (Table 7).

Personal experience with COVID-19 (PC4) was a significant predictor of GAD-7 in 9 countries – Armenia, Canada, India, Indonesia, Malaysia, Russia, Saudi Arabia, Turkey, and the United States. The citizens from these countries who fell ill themselves or had someone infected within their close environment had higher ratings of anxiety (Table 7).

The results of a regression analysis with SAI for each country are presented in Table 8. Again, we excluded Tanzania from the analysis, as some questions were not completed in this country.

**TABLE 8:** Regression analysis for the factors predicting anxiety (SAI) in each country.

Country	*R* ^2^	Predictor	*B*	*SE*	Beta	*t*	*p*
Armenia	0.301	PC1	5.776	2.067	0.448	2.795	**0.009**
		PC2	2.635	2.292	0.190	1.150	0.260
		PC3	0.556	2.123	0.044	0.262	0.795
		PC4	5.780	2.707	0.346	2.135	**0.042**
Belarus	0.070	PC1	1.047	0.722	0.080	1.450	0.148
		PC2	–0.055	0.852	–0.003	–0.064	0.949
		PC3	–2.614	0.654	–0.218	–3.997	**<0.001**
		PC4	0.796	0.542	0.078	1.468	0.143
Brazil	0.065	PC1	3.225	0.833	0.175	3.871	**<0.001**
		PC2	–0.159	0.767	–0.009	–0.207	0.836
		PC3	–1.279	0.528	–0.109	–2.425	**0.016**
		PC4	0.734	0.331	0.095	2.219	**0.027**
Bulgaria	0.036	PC1	1.521	0.611	0.140	2.490	**0.013**
		PC2	–0.723	0.845	–0.048	–0.856	0.393
		PC3	–0.731	0.659	–0.062	–1.110	0.268
		PC4	1.062	0.792	0.075	1.342	0.181
Canada	0.026	PC1	–0.659	0.481	–0.066	–1.370	0.171
		PC2	0.007	0.509	0.001	0.015	0.988
		PC3	–1.785	0.583	–0.149	–3.064	**0.002**
		PC4	0.866	0.313	0.119	2.764	**0.006**
Croatia	0.064	PC1	2.772	0.810	0.203	3.421	**0.001**
		PC2	–1.449	1.043	–0.084	–1.389	0.166
		PC3	–1.343	0.768	–0.105	–1.748	0.082
		PC4	0.563	0.966	0.035	0.583	0.561
Hungary	0.097	PC1	2.502	0.800	0.199	3.130	**0.002**
		PC2	–2.567	1.008	–0.161	–2.547	**0.012**
		PC3	–2.038	0.856	–0.152	–2.382	**0.018**
		PC4	–1.374	0.797	–0.108	–1.723	0.086
India	0.128	PC1	1.916	0.468	0.200	4.091	**<0.001**
		PC2	–0.667	0.527	–0.064	–1.264	0.207
		PC3	–1.833	0.547	–0.173	–3.351	**0.001**
		PC4	1.642	0.483	0.164	3.401	**0.001**
Indonesia	0.106	PC1	2.594	0.390	0.209	6.656	**<0.001**
		PC2	–0.564	0.435	–0.042	–1.296	0.195
		PC3	–2.503	0.463	–0.174	–5.402	**<0.001**
		PC4	1.687	0.382	0.138	4.423	**<0.001**
Iran	0.011	PC1	–0.125	0.190	–0.039	–0.654	0.513
		PC2	–0.146	0.243	–0.035	–0.599	0.549
		PC3	–0.006	0.222	–0.002	–0.026	0.979
		PC4	0.175	0.112	0.091	1.564	0.119
Iraq	0.044	PC1	1.120	0.939	0.097	1.193	0.235
		PC2	–0.644	1.107	–0.049	–0.581	0.562
		PC3	–1.410	0.804	–0.149	–1.753	0.082
		PC4	0.630	0.743	0.069	0.847	0.398
Italy	0.063	PC1	2.219	0.739	0.187	3.001	**0.003**
		PC2	–1.172	0.890	–0.083	–1.317	0.189
		PC3	–0.886	0.889	–0.063	–0.997	0.320
		PC4	0.944	0.574	0.102	1.646	0.101
Jordan	0.027	PC1	1.116	0.421	0.128	2.652	**0.008**
		PC2	–1.153	0.864	–0.066	–1.335	0.183
		PC3	–0.767	0.640	–0.060	–1.200	0.231
		PC4	1.420	0.965	0.069	1.471	0.142
Malaysia	0.027	PC1	2.142	0.456	0.153	4.703	**<0.001**
		PC2	–0.788	0.586	–0.042	–1.344	0.179
		PC3	–1.040	0.865	–0.039	–1.202	0.229
		PC4	0.458	0.652	0.021	0.702	0.483
Nigeria	0.200	PC1	2.501	0.459	0.277	5.443	**<0.001**
		PC2	3.607	0.766	0.242	4.709	**<0.001**
		PC3	–2.663	0.540	–0.253	–4.934	**<0.001**
		PC4	1.097	0.650	0.086	1.687	0.093
Pakistan	0.079	PC1	2.662	0.510	0.230	5.215	**<0.001**
		PC2	–0.155	0.673	–0.010	–0.231	0.818
		PC3	–1.570	0.549	–0.129	–2.859	**0.004**
		PC4	0.430	0.437	0.043	0.982	0.326
Romania	0.035	PC1	1.839	0.720	0.155	2.554	**0.011**
		PC2	–0.452	0.955	–0.029	–0.473	0.636
		PC3	–1.555	0.966	–0.100	–1.611	0.108
		PC4	0.263	0.658	0.024	0.399	0.690
Russia	0.045	PC1	1.075	0.259	0.097	4.151	**<0.001**
		PC2	–1.100	0.341	–0.076	–3.222	**0.001**
		PC3	–1.378	0.250	–0.127	–5.512	**<0.001**
		PC4	1.250	0.337	0.084	3.704	**<0.001**
Saudi Arabia	0.082	PC1	1.797	0.547	0.159	3.285	**0.001**
		PC2	–1.729	1.005	–0.082	–1.721	0.086
		PC3	–3.287	0.917	–0.173	–3.585	**<0.001**
		PC4	1.309	0.816	0.077	1.604	0.109
Turkey	0.033	PC1	0.912	0.141	0.093	6.457	**<0.001**
		PC2	–1.174	0.120	–0.151	–9.777	**<0.001**
		PC3	–0.762	0.113	–0.103	–6.733	**<0.001**
		PC4	0.170	0.135	0.018	1.261	0.208
Thailand	0.028	PC1	0.239	0.509	0.028	0.470	0.639
		PC2	–1.855	0.678	–0.166	–2.736	**0.007**
		PC3	0.170	0.953	0.011	0.179	0.858
		PC4	0.147	0.617	0.014	0.238	0.812
United States	0.072	PC1	1.819	0.610	0.125	2.984	**0.003**
		PC2	–0.624	0.726	–0.036	–0.860	0.390
		PC3	–3.245	0.626	–0.200	–5.181	**<0.001**
		PC4	0.859	0.388	0.086	2.213	**0.027**

*The bold values show significant association.*

In the case of the first factor (PC1), 17 countries demonstrated a positive association between anxiety and personal awareness of the threat of COVID-19 – Armenia, Brazil, Bulgaria, Croatia, Hungary, India, Indonesia, Italy, Jordan, Malaysia, Nigeria, Pakistan, Romania, Russia, Saudi Arabia, Turkey, and the United States. Notably, in the case of the SAI scale Malaysia had a positive association of anxiety and PC1, contra GAD-7 ratings (Table 8).

The second factor (PC2) was a significant predictor of anxiety SAI only in 5 countries – Hungary, Nigeria, Russia, Turkey, and Thailand. More anxious people in four of these countries (Hungary, Russia, Turkey, and Thailand) were those who did not believe in officially undertaken measures and thought that measures were introduced too late, as well as those who felt more hostile and suspicious reaction to foreigners. On the contrary, in Nigeria this association was positive (Table 8).

Personal trust in official sources (PC3) was a significant predictor of anxiety SAI in 12 countries – Belarus, Brazil, Canada, Hungary, India, Indonesia, Nigeria, Pakistan, Russia, Saudi Arabia, Turkey, United States. The level of anxiety decreased with trust in official sources. The high level of anxiety in these countries was associated with low personal trust in government and official sources (Table 8).

Personal experience with COVID-19 (PC4) was a significant predictor of anxiety SAI in 7 countries – Armenia, Brazil, Canada, India, Indonesia, Russia, and the United States. People with personal experience of coronavirus reported higher ratings of anxiety (Table 8).

## Discussion

The results of the current cross-cultural study revealed differences in anxiety variables between the participants from 23 countries during the first wave of COVID-19, as well as differences in association with the personal awareness of the threat of COVID-19, personal reaction toward officially undertaken measures and attitudes to foreigners, personal trust to official sources and personal experience with COVID-19.

### Country Differences in Anxiety Scales

**FIGURE 5:**
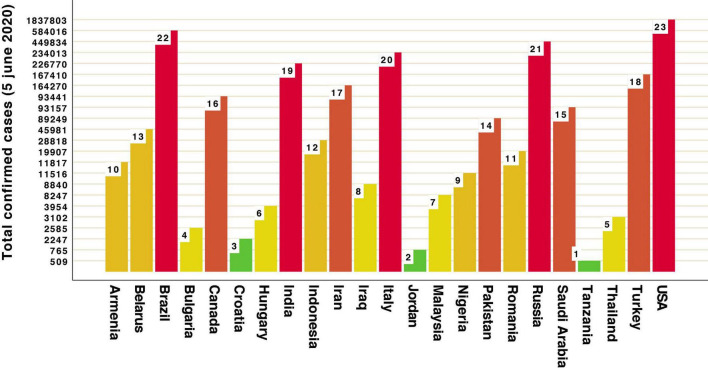
Total confirmed cases of COVID-19 across 23 countries (WHO data).

Our data revealed that the highest GAD-7 scores during restrictions and lockdown of the first wave of COVID-19 were in participants from Iraq, Canada, Brazil, Croatia, Italy (when looking at the GAD-7 scale) and Brazil, Italy, Iran (SAI scale). Most of these countries rated highest in the number of total confirmed cases of COVID-19 (Figure 5). Lowest anxiety scores were in participants from Malaysia, Indonesia, Thailand (as measured by GAD-7), Romania and Nigeria (as measured by SAI). All are rated as countries with medium numbers of total confirmed cases (Figure 5). These results may be discussed in line with cultural dimensions, such as collectivism/individualism or tightness/looseness. Collectivistic societies put more emphasis on group interest over personal interests and enjoyment, which is in contrast to individualistic societies (Hofstede, 2001). The dimension of cultural tightness-looseness refers to the strength of cultural norms: tight culture (e.g., Pakistan, Singapore, South Korea, and China) allows little room for individual liberty and poses high censuring pressure, whereas a loose culture provides members more room for discretion (Gelfand et al., 2011). The data presented by Kowal et al. (2020), revealed no association along the continuum of individualism–collectivism and anxiety. In this study participants with the high ratings of anxiety were from countries which scored high on individualism and looseness indexes (Canada, Italy, United States, Brazil) (Hofstede, 2001; Gelfand et al., 2011). In contrast, the least anxious ratings were obtained for respondents from collectivistic countries (Thailand, Indonesia, Malaysia, and Nigeria). Other authors stated that Brazil, Colombia, and the United States demonstrated higher levels of anxiety compared to Israel, Germany, and Norway (Mækelæ et al., 2020). The study conducted on 54 nations tested how the cultural variations in individualism and tightness affected the containment of COVID-19 during a 30-day period of government intervention (restrictions and measures to mitigate or stop the virus) (Cao W. et al., 2020). It demonstrated significant relationships between cultural variables and national performance in slowing the spread of the coronavirus, measured by the three tightness–looseness indexes (namely, changes in the prevalence rate, crude mortality rate and case fatality rate – and their interaction). Loose and individualistic nations experienced higher rates of increases in infected cases and deaths than tight and collectivistic ones (Cao W. et al., 2020).

### Anxiety and Personal Awareness of the Threat of COVID-19

High levels of anxiety were significantly correlated with high levels of personal awareness of the threat of COVID-19 in our study in both anxiety scales for a majority of countries. High level of GAD-7 anxiety was associated with more personal awareness of the threat of COVID-19 in Croatia, Hungary, India, Jordan, Malaysia, Pakistan, Romania, Russia, Saudi Arabia, and the United States, but not in Malaysia. Personal awareness of the threat of COVID-19 was a significant predictor of SAI anxiety in Armenia, Brazil, Bulgaria, Croatia, Hungary, India, Indonesia, Italy, Jordan, Malaysia, Nigeria, Pakistan, Romania, Russia, Saudi Arabia, Turkey, and the United States. Past research on the impact of the epidemics on psychological health has shown that a fear of infection has been a good predictor of increased stress (Cava et al., 2005; Desclaux et al., 2017; Brooks et al., 2020; Luo et al., 2021). The same is true for recent studies; for example, in Jordan fear toward the COVID-19 outbreak correlated with downloaded applications to trace COVID-19 cases, and many respondents mentioned that they were very afraid of the COVID-19 virus and were feeling uncomfortable thinking about it or when watching news and stories related to the pandemic on social media (Abuhammad et al., 2021). Another study reported that 72% of Indian respondents had concerns for themselves and their loved ones during the COVID-19 pandemic (Roy et al., 2020). In Italy, a collective ritual has been consolidating during the first phases of the pandemic, as evidence of this threat: listening on a daily basis to civil protection’s announcements of the number of deaths, contagions, and people who had to be hospitalized or even admitted to intensive care units. Some compared this ritual to that of tuning to BBC radio during the Second World War (Cipolletta and Ortu, 2021, p. 280). Coronavirus anxiety positively correlated with fear about coronavirus in an online survey of 398 adult Amazon MTurk workers in the United States (Lee et al., 2020). Recent data suggest, however, that accurate public risk perceptions are critical to effectively managing public health risks (Dryhurst et al., 2020). Particularly, it was found that higher collective efficacy beliefs reduced risk perceptions about COVID-19 in Spain, Japan, Mexico, the United Kingdom, and the United States (Dryhurst et al., 2020). Hence, it may be concluded, that the factor of awareness of the real danger of a pandemic associates with other significant factors, especially with the trust in official sources (government, official mass media, laws and restrictions), the reaction on taken measures and the personal experiences of COVID-19. Lastly, a systematic review and meta-analysis of fear of COVID-19 across 44 articles with a sample size of 52,462 showed the mean of fear of COVID-19 was high around the world (Luo et al., 2021).

### Anxiety and Personal Reaction Toward Officially Undertaken Measures and Attitudes to Foreigners

A high level of GAD-7 anxiety in our study was significantly correlated with low levels of personal reaction toward officially undertaken measure (did not believe in officially undertaken measures and think that measures were introduced too late) and attitudes to foreigners (felt more hostile and suspicious reaction to foreigners) in two countries: Russia and Hungary. In the case of the SAI scale this factor significantly predicted anxiety level in five countries – Russia and Hungary, as well as Nigeria, Turkey, and Thailand. More anxious people in all these countries (excluding Nigeria) were those who did not believe in officially undertaken measures and think that measures were introduced too late, as well as those who felt more hostile and suspicious reaction to foreigners. In the case of Russia, this may be interpreted in terms of the high levels of power distance (extent to which the less powerful members of organizations and institutions (like the family) accept and expect that power is distributed unequally) found in earlier works (Javidan and Dastmalchian, 2009; Fedenok and Burkova, 2020) on the one hand, and by spatial proximity to China and the common border on the other hand. According to other findings from Brazil, Colombia, Germany, Israel, Norway and US, more worried and stressed people showed less trust in authority, and expressed high pessimism related to governmental ability to control the outbreak (Mækelæ et al., 2020).

Previous experience with epidemics, as well as current data, suggest that anxiety and fear related to infection may lead to various acts of discrimination (McCauley et al., 2013; Monson, 2017; Chui, 2020; Ren et al., 2020). For example, it is known that people from Wuhan were targeted and blamed for the COVID-19 outbreak by other Chinese people, and the Chinese people in the whole have been stigmatized internationally in media, as the COVID-19 has been entitled as the “China virus”/the “Wuhan virus”/the “New Yellow Peril” (Chui, 2020; Ren et al., 2020). Dating back to 2014, during Ebola outbreak, people of African descent were intensively discriminated outside Africa (Monson, 2017), and during the 2009 H1N1 flu outbreak in the United States the Mexicans and migrant workers were subjected to discrimination (McCauley et al., 2013). Since the spread of COVID-19 in January 2020 the United Kingdom and the United States have seen an increase in reports of violence and hate crimes against people of Asian descent and an overall rise in anti-Chinese sentiments (Usher et al., 2020). Misinformation plays an important role in this discrimination and government and health officials should be aware of this problem, and be able to help protect the vulnerable and endangered groups of population. Perceived mixed and unclear messaging from state authorities can also result in public confusion and fear (Han et al., 2018). Research conducted in Poland and the United Kingdom showed a positive relationship between media exposure in the both countries, and prejudice against four foreign nationalities (Sorokowski et al., 2020). The same is true, with obviously negative reactions toward Italians in Europe and United States, i.e., the nations struggling with the most severe COVID-19 outbreak at the time of the study (Sorokowski et al., 2020).

### Anxiety and Personal Trust in Official Sources

Personal trust in official sources was a significant predictor of GAD-7 in 13 countries from our study, including Belarus, Brazil, Croatia, India, Indonesia, Iran, Jordan, Nigeria, Pakistan, Russia, Saudi Arabia, Turkey, and the United States, and was a predictor of SAI anxiety level in 12 countries, including Belarus, Brazil, Canada, Hungary, India, Indonesia, Nigeria, Pakistan, Russia, Saudi Arabia, Turkey, and the United States. Respondents from these countries who did not trust official sources exhibited higher anxiety scores. Past studies of the 2001 foot and mouth disease and the 2009 swine flu showed that perceptions of government action were associated with judgments of trust (Poortinga et al., 2004; van der Weerd et al., 2011; Dryhurst et al., 2020). A study of social distancing in the context of the coronavirus pandemic conducted among Russian-speaking respondents living or staying in various countries at the time of the outbreak and spread of the coronavirus also demonstrated that individual behavior in the context of the COVID-19 pandemic has been affected by country of residence, trust in authorities, awareness of the prescribed rules of behavior, and cultural norms and traditions (Fedenok and Burkova, 2020). Moreover, these factors affected both the perception of the situation and the implementation of the authorities’ recommendations. According to earlier findings, trust and beliefs in the effectiveness of the adopted restrictions contribute to the observance of the recommended preventive measures of behavior (Maddux and Rogers, 1983). It should also be mentioned, that variations in reactions of political leaders around the world in the time of the COVID-19 outbreak not only affected the country infection rate, but also the rate of public trust in leaders and people’s responses to the pandemic (Han et al., 2020; Mækelæ et al., 2020; Wilson, 2020).

### Anxiety and Personal Experience With COVID-19

High levels of anxiety were significantly correlated with personal experience with COVID-19. It was a significant predictor of GAD-7 in 9 countries – Armenia, Canada, India, Indonesia, Malaysia, Russia, Saudi Arabia, Turkey, and the United States; and SAI in seven countries – Armenia, Brazil, Canada, India, Indonesia, Russia, and the United States. People from countries where citizens had been familiar with a new coronavirus or other pandemic infections revealed the higher ratings of anxiety. These findings are generally consistent with the data of another cross-cultural study conducted in the United Kingdom, United States, Australia, Germany, Spain, Italy, Sweden, Mexico, Japan, and South Korea that people with direct personal experience of infection turned to perceive the risk of COVID-19 significantly more seriously (Dryhurst et al., 2020). A study of the impact of COVID-19 experiences and associated stress showed that COVID-19 experiences were consistently associated with higher odds of probable anxiety and depression diagnoses and predicted large proportions of variance (*R*^2^ ≥ 30%) in anxiety, depression, and functional impairment, with the worst outcomes associated with a confirmed COVID-19 diagnosis and death of relatives and close friends (Gallagher et al., 2020). Current research has documented elevated symptoms of depression, anxiety, and stress among those who have contracted COVID-19 (Yao et al., 2020).

## Conclusion

The results presented in this paper revealed the general increase of anxiety during the first wave of the COVID-19 pandemic, as well as cross-cultural variations in the level of anxiety observed. Along with the findings from other scholars (Berta et al., 2020; Brooks et al., 2020; Cao C. et al., 2020; Chen et al., 2020; Kowal et al., 2020; Mækelæ et al., 2020; van Bavel et al., 2020; Rodríguez et al., 2021; etc.), as well as our previous data (Burkova et al., 2021), we conclude, that feelings of anxiety as well as being stressed is a normal reaction of the human psyche in the face of global threat. Age, sex, education, living conditions, having family, economic status, access to internet and mobile communications are among the universal factors potentially affecting personal anxiety during pandemic (Burkova et al., 2021; Butovskaya et al., 2021; Semenova et al., 2021). Individuals reacted differently to a health-threatening condition such as COVID-19, based on their own illness behavior - this concept to describe the different ways in which individuals may perceive, evaluate, and react to certain physical symptoms (Mechanic, 1995; Cosci and Guidi, 2021). Illness behavior represents the result of different interacting variables, including individual, social, and cultural determinants. In our research cross-cultural differences in levels of anxiety, as well as the proportion of citizens being stressed by the pandemic, vary due to a number of factors, including personal comprehension of the danger and understanding of its consequences, trust in the government, hostility to foreigners, information presented by media, and previous experience with pandemics.

The developmental trajectory of the epidemic situation in the countries, investigated during the first wave, provided additional sources of information. Our data from 23 countries showed that such cultural dimensions as individualism/collectivism, power distance and looseness/tightness may function as protective adaptive mechanisms against the development of anxiety disorders in a pandemic situation (Burkova et al., 2021). Countries with high distance to power, strict governmental restrictions and quarantine measures, high availability of medical services, and afterward with access to COVID-19 vaccines and effective state programs for the vaccination of citizens, were generally doing better in terms of the number of infected and deaths per capita. Whether country-level anxiety has been fluctuating in accordance with positive or negative changes in this respect remains to be tested in the future. This study provides interesting findings that may help to plan tailored interventions aimed to reduce anxiety related to COVID-19, considering cultural differences. The varying psychological responses observed during the COVID-19 pandemic can be effectively subsumed under the conceptual framework of illness behavior. It may substantially impact on the use of healthcare services, treatment adherence, and self-management behaviors.

### Limitation

Limitations of the current study include the disproportionate representation of women to men. Additionally, it is important to acknowledge that while the overall sample included over 15,000 participants, the representation in some countries (i.e., Armenia, Iraq) was quite low, which limits our ability to examine within-country differences. In addition, the magnitude of changes in anxiety and depression symptoms will vary under political and cultural situation in each country (for example, in this study, the level of anxiety in Iraq was very high, and this was a consequence not only of COVID-19, but also of a difficult political situation in the country). Differences in the roles of men and women across cultures have not been accounted in frame of this study, but future research needs to further explore these relationships to better understand gender differences in pandemic responses. Another consideration is that participation in this study was limited to those with a stable internet connection (to complete the questionnaire), which precluded participation from those without this access. We did not measure countries’ policies relating to COVID-19 and mortality rates, which may also be an important predictor of anxiety increase. Because the situation with COVID-19 is rapidly changing, we anticipate that some of the things we will consider may seem plausible today but might not be relevant tomorrow.

## Data Availability Statement

The raw data supporting the conclusions of this article will be made available by the authors, without undue reservation.

## Ethics Statement

The studies involving human participants were reviewed and approved by Scientific Council of the Institute of Ethnology and Anthropology of the Russian Academy of Sciences. The patients/participants provided their written informed consent to participate in this study.

## Author Contributions

MB and VB: conceptualization, data analysis, writing-original draft preparation, visualization, and project administration. MB, VB, and AR: methodology. VB: data curation. All authors contributed to the data collections, resources, read and agreed to the published version of the manuscript.

## Conflict of Interest

The authors declare that the research was conducted in the absence of any commercial or financial relationships that could be construed as a potential conflict of interest.

## Publisher’s Note

All claims expressed in this article are solely those of the authors and do not necessarily represent those of their affiliated organizations, or those of the publisher, the editors and the reviewers. Any product that may be evaluated in this article, or claim that may be made by its manufacturer, is not guaranteed or endorsed by the publisher.

## Footnotes

1 In this research we used term “culture” as social norms and moral institutions, social distancing rules and social network structure of society.

2 WHO Weekly operational update on COVID-19 on 5 June 2020 https://cdn.who.int/media/docs/default-source/sri-lanka-documents/20200605-covid-19-sitrep-137.pdf?sfvrsn=a13df572_2.
